# The complete chloroplast genome of *Periploca forrestii* (Apocynaceae), a traditional Chinese medicinal plant

**DOI:** 10.1080/23802359.2022.2049459

**Published:** 2022-03-09

**Authors:** Jin-lan Long, Na Zhang, Yuan Wu, Yong-pin Zhang, Zhi-kun Wu

**Affiliations:** Department of Pharmacy, Guizhou University of Traditional Chinese Medicine, Guiyang, China

**Keywords:** Complete chloroplast genome, phylogenetic analysis, *Periploca forrestii*

## Abstract

*Periploca forrestii* Schltr. is a traditional medicine plant in southwestern China. In this study, we characterize the complete chloroplast (cp) genome of *P. forrestii* based on next-generation sequencing. The cp genome is 154,140 bp in size with an overall GC content 38.2%, including a large single-copy (LSC) region (84,941 bp), a small single-copy (SSC) region of 17,619 bp, and two inverted repeats (IRs) regions, each of 25,790 bp. A total of 130 genes (85 protein-coding genes, 8 ribosomal RNA (rRNA) genes, and 37 transfer RNA (tRNA genes)) are annotated in the whole chloroplast genome, containing 113 unique genes (79 unique CDSs, 30 unique tRNAs, and 4 unique rRNAs). The phylogenetic analysis indicated that *P. forrestii* formed a monophyletic clade with the same genus plant *P. sepium*, showing that they have close relationship. The complete chloroplast genome of *P. forrestii* provides valuable genomic information for the phylogeny, molecular identification and sustainable utilization of this species.

The genus *Periploca* L. traditionally was belonging to Periplocoideae of Asclepiadaceae (Li et al. 1995), which is now included into Apocynaceae (APG IV 2016). There are about 10 species in *Periploca*, mainly distributed in temperate Asia, southern Europe, and tropical Africa (Li et al. 1995). The number of species of *Periploca* in China is uncertain, in Browicz’s monograph of the genus, which recognized in China only two species, one with three subspecies (Browicz 1966); in Flora Reipublicae Popularis Sinicae, the species is four (Jiang and Li 1977); but in Flora of China, the number is five (Li et al. 1995); and in recent review on this genus, the species is seven in China, with two new species (Huang et al. 2019), therefore the boundaries of the inter-species is not clear in this genus in China to date.

*Periploca forrestii* Schlechter 1913 is a traditional medicine plant in southwestern China, mainly distributed in Guangxi, Guizhou, Qinghai, Sichuan, Xizang, and Yunnan, grows in thickets, sparse montane woods under the altitude of 2000 m. The stems or the whole plant of *P. forrestii*, are widely used by the Miao nationality in Guizhou, China, to treat many diseases, such as rheumatic arthritis, traumatic injury, stomachache, dyspepsia, and amenorrhea (Huang et al. 2019). In this study, we report the first chloroplast (cp) genome of *P. forrestii*, which will provide valuable genomic information for the phylogeny, molecular identification and sustainable utilization of this species.

The fresh leaves of *P. forrestii* were collected from a wild population (106° 40′30″ E, 26^°^26′34″ N) in Huaxi district, Guizhou Province, China. The specimen was deposited at the herbarium of Guizhou University of Traditional Chinese Medicine (Cheng-gang Hu, 2274547063@qq.com) under the voucher number LJL20200403. The total genomic DNA was isolated following a modified CTAB protocol (Doyle 1991). The purified genomic DNA was sheared into c. 300 bp fragments to construct a paired-end (PE) library according to the Nextera XT sample preparation procedures (Illumina, San Diego, CA), and the PE reads of 150 bp was generated by HiSeq X-Ten sequencer (Illumina, San Diego, CA). In total, 3.18 Gb of raw sequence data were obtained. We assembled the complete chloroplast genome using NOVOplasty v2.7.2 (Dierckxsens et al. 2017), and used Geneious v 8.0.2 software to annotate the chloroplast genome assembled (Kearse et al. 2012). The annotated chloroplast genome of *P. forrestii* was deposited into GenBank with the accession number MZ557568.

The total length of the chloroplast genome is 154,140 bp, with an overall GC content of 38.2%. This cp genome presents a typical quadripartite structure, including a large single-copy (LSC) region (84,941 bp), a small single-copy (SSC) region of 17,619 bp, and two inverted repeats (IRs) regions, each of 25,790 bp. A total of 130 genes (85 protein-coding genes, 8 ribosomal RNA (rRNA) genes, and 37 transfer RNA (tRNA genes)) are annotated in the whole chloroplast genome, containing 113 unique genes (79 unique CDSs, 30 unique tRNAs, and 4 unique rRNAs respectively).

The phylogenetic relationship between *P. forrestii* and related taxa was inferred base on 16 complete chloroplast genome sequences of Apocynaceae species and *Coleus xanthanthus* as outgroup, these sequences were obtained from GenBank and aligned by MAFFT (Katoh and Standley 2013). We constructed the maximum likelihood (ML) tree using IQ-TREE v1.6.10 (Nguyen et al. 2015) and performed it base on TVM + F+R2 model according to Bayesian information criteria using ModelFinder (Kalyaanamoorthy et al. 2017). Ultrafast bootstrap (UFBoot) was used to test branch supports (Hoang et al. 2018) and SH-like approximate likelihood ratio with 10,000 bootstrap replicates (Figure 1). The phylogenetic results indicated that the same genus plants *P. forrestii* and *P. sepium* formed a monophyletic clade with 100% bootstrap value, showing that they have close relationship. This published *P. forrestii* chloroplast genome will provide useful information for molecular identification of close species of *Periploca*, and also for phylogenetic and evolutionary studies in Apocynaceae.

**Figure 1.**
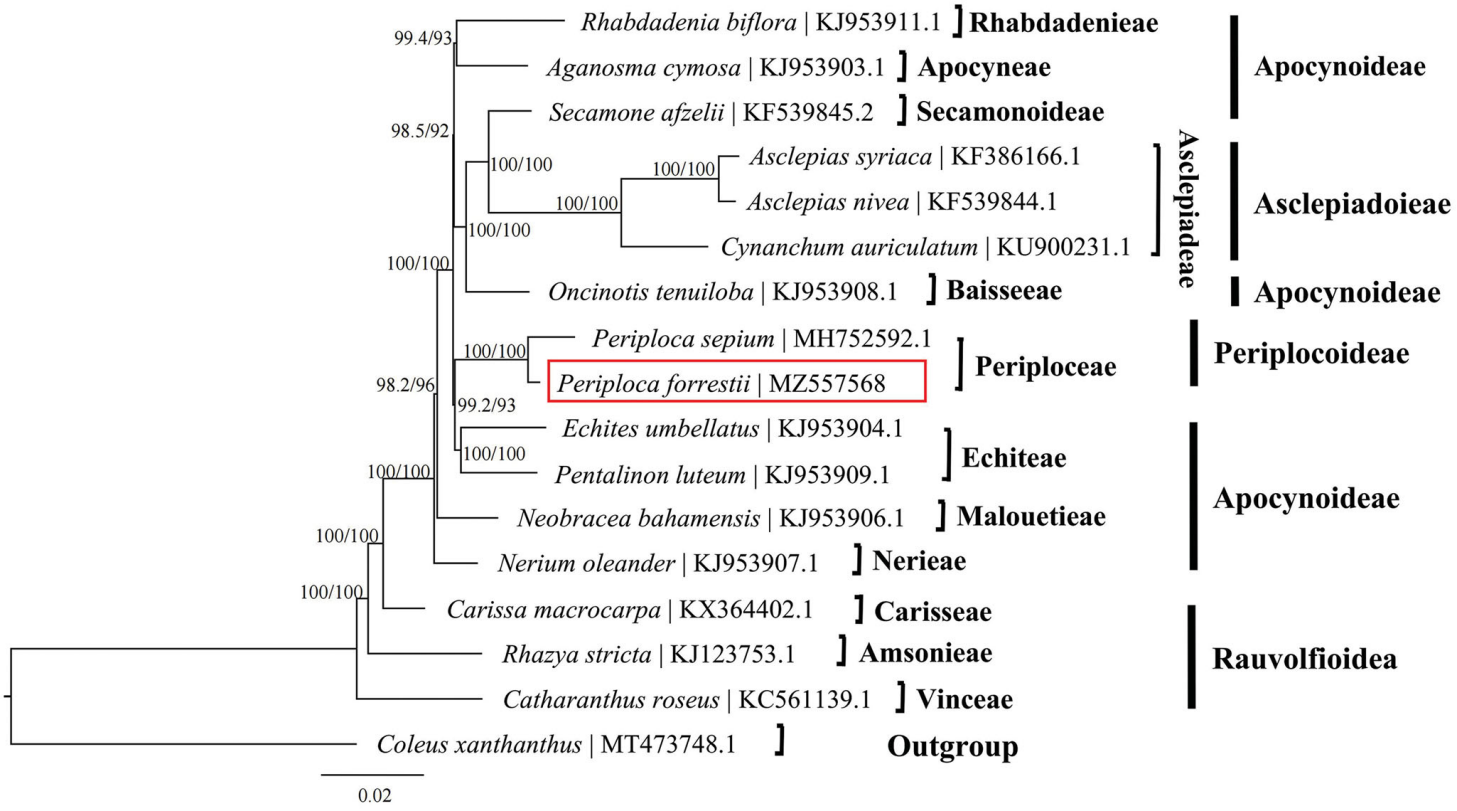
The maximum likelihood (ML) tree based on the complete chloroplast genome sequences of *Periploca forrestii* and related taxa, branch supports values are reported as SH-aLRT/UFBoot, the accession number of GenBank for each species is listed in the figure.

## Data Availability

The genome sequence data that support the findings of this study are openly available in GenBank of NCBl at (https://www.ncbi.nlm.nih.gov/) under the accession no.MZ 557568.The associated **BioProject** **SRA**and**Bio-Sample** numbers are PRJNA747848, SRR15184071 and SAMN20297375, respectively.
